# When is reacquisition necessary due to high extra-cardiac uptake in myocardial perfusion scintigraphy?

**DOI:** 10.1186/2191-219X-3-20

**Published:** 2013-03-25

**Authors:** Allan Johansen, Milan Lomsky, Oke Gerke, Lars Edenbrandt, Lena Johansson, Gunhild Hansen, Birgitte Jensen, Maria Sallerup Reid, Lise-Lott Johansson, Camilla Olofsson, David Minarik, Karin Nyström, Per Wollmer, Elin Trägårdh

**Affiliations:** 1 Department of Nuclear Medicine, Odense University Hospital, Odense, Denmark; 2 Department of Molecular and Clinical Medicine, Clinical Physiology, Sahlgrenska University Hospital, Gothenburg, Sweden; 3 Centre of Health Economics Research, Department of Business and Economics, University of Southern Denmark, Odense, Denmark; 4 Nuclear Medicine Unit, Skåne University Hospital, Lund University, Malmo, Sweden; 5 EXINI Diagnostics AB, Lund, Sweden

**Keywords:** Myocardial perfusion imaging, Quality assessment, Image interpretation, Extra-cardiac uptake, Ischemic heart disease

## Abstract

**Background:**

Technetium-labeled agents, which are most often used for assessing myocardial perfusion in myocardial perfusion scintigraphy (MPS), are cleared by the liver and excreted by the biliary system. Spillover from extra-cardiac activity into the myocardium, especially the inferior wall, might conceal defects and lower the diagnostic accuracy of the study. The objective was to determine rules of thumb for when reacquisition is useful due to high extra-cardiac uptake, i.e., when interpretation of the studies was affected by poor image quality.

**Methods:**

Patients admitted to MPS at any of the three study sites, who also underwent a reacquisition due to high extra-cardiac uptake were included. Image quality was assessed by ten technologists on a scale ranging from 1 to 5. Interpretations regarding the presence/absence of ischemia/infarction, including the certainty of the diagnosis, were made by three physicians.

**Results:**

There was a statistically significant increase in image quality between the first and the repeated acquisition (1,256 cases of increased quality at the repeated study (66%), 134 cases of decreased quality at the repeated study (7%), 510 cases of unchanged quality (27%) *P* < 0.0001). The number of equivocal studies, interpreted by physicians, decreased when evaluating the repeated studies compared to the first studies for all physicians, both for the interpretations of ischemia and for infarction. Receiver operating characteristic analyses revealed that for both endpoints (ischemia, infarction) and all physicians, the optimal cutoff point for performing a reacquisition was between quality categories 2 and 3.

**Conclusion:**

This study indicates that repeat acquisition is useful when (1) the intensity of the extra-cardiac uptake is equal to or higher than the cardiac uptake when there is no separation between the extra-cardiac uptake and the inferior cardiac wall and (2) when the intensity of the extra-cardiac uptake is higher than the cardiac uptake when there is a separation between the extra-cardiac uptake and the inferior wall of less than one cardiac wall.

## Background

Myocardial perfusion scintigraphy (MPS) is widely regarded as a clinically useful noninvasive imaging method for the diagnosis of suspected coronary artery disease, identification of culprit lesions and risk assessment [1-4]. Technetium-labeled agents, which are most often used for assessing myocardial perfusion, are cleared by the liver and excreted by the biliary system. Activity in the subdiaphragmatic organs can interfere with evaluation of perfusion in two general ways. Spillover from the extra-cardiac activity into the myocardium, especially the inferior wall, might conceal defects and lower the diagnostic accuracy of the study. The spillover is a result of the limited spatial resolution of the camera system and scattered photons that are detected in the myocardial area but emanate from the extra-cardiac activity. High extra-cardiac activity can also result in decreased activity in the adjacent myocardium, if filtered back-projection (FBP) is used [5]. This latter artifact does not occur if iterative reconstruction such as ordered subset expectation maximization (OSEM) reconstruction is used.

The artifacts due to high extra-cardiac activity are patient dependent, and their impact on the image interpretation is difficult to predict. A common way to handle problems regarding extra-cardiac activity in clinical routine is to perform repeat acquisitions when the extra-cardiac activity is assumed to have cleared from the vicinity of the heart. There is limited clinical evidence as to the usefulness of repeat acquisitions and when they should be performed.

The objective of the study was to determine rules of thumb for when reacquisition is useful due to high extra-cardiac uptake adjacent to the heart, i.e., when interpretation of the studies is affected by poor image quality. In this study, only extra-cardiac activity resulting in spillover was investigated.

## Methods

The study population includes patients who underwent a MPS at Sahlgrenska University Hospital, Gothenburg, Sweden, Skåne University Hospital, Malmö, Sweden, or Odense University Hospital, Odense, Denmark*.* Only patients who had a reacquisition according to clinical routine due to high extra-cardiac uptake were included. Mean time between the first and the repeat acquisition was 88 min (standard deviation 37 min). In cases of several reacquisitions on the same patient, only one was included.

**Figure 1:**
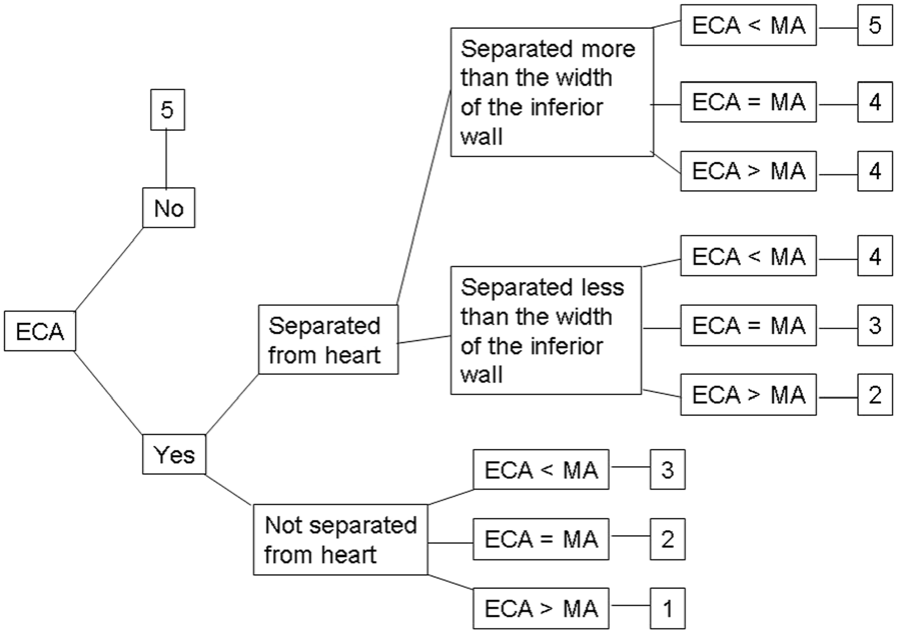
**Instructions to technologists for how to grade quality of the images.** ECA, extra-cardiac uptake; MU, myocardial uptake.

**Figure 2:**
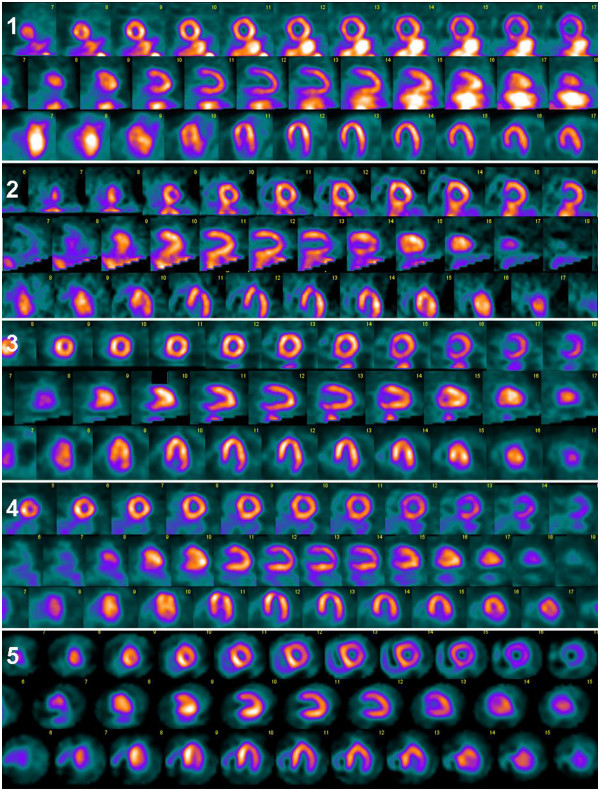
Examples of the grading of image quality (grading 1 to 5).

All the images were presented to three or four technologists at each site (three from Gothenburg and Odense, respectively, and four from Malmö), using a modified version of EXINI heart^TM^ (EXINI Diagnostics, Lund, Sweden), where only slice images and polar plots were provided. No quantification (scoring values, blackouts, etc.) was presented. In total, 1,900 evaluations were performed (92 patients from Gothenburg, 25 patients from Malmö, 74 patients from Odense). No clinical information about the patient or the study was available to the technologists. The technologists judged image quality on a scale ranging from 1 to 5; 1 being worst (Figure 1), and judged the first and the repeated acquisition images separately and in random order. The judgment was based on the distance between the extra-cardiac activity and the heart wall as well as on the relative intensities of extra-cardiac activity and myocardium, which was determined by visual assessment. Illustrative examples are shown in Figure 2. This scale, and examples for each grade, had been agreed on at a joint meeting at the start of the study.

For the evaluations made by physicians, all patients who did not have a corresponding stress or a rest study to the first or reacquisition available were excluded. Sixteen new Gothenburg patients were also added in order to increase the statistical power, which were not included in the quality evaluation performed by technologists. In total, 112 patients from Gothenburg, 5 patients from Malmö, and 17 patients from Odense were included. Three experienced physicians, one from each study center, independently classified the studies (134 patients × 2 studies) with regard to the presence/absence of ischemia/infarction in grades from 1 to 5 (1 = no ischemia/infarction, 2 = probably no ischemia/infarction, 3 = equivocal, 4 = probable ischemia/infarction, and 5 = certain ischemia/infarction). The physicians were blinded to any patient data, except gender, including which studies belonged to the same patient and if the study was the first acquisition or the reacquisition. They did not know if the extra-cardiac uptake decreased at the reacquisition. Only slice images and polar plots were presented, without quantification such as scoring values. In the evaluation of the interrelationships between the technologists’ quality assessment and the physicians’ confidence in rating myocardial ischemia and infarction the material consisted of in total 111 patients, which were evaluated by all ten technologists and three physicians. Of these, 91 patients were from Gothenburg, 4 from Malmö, and 16 from Odense.

The study was performed in accordance with the principles of the Declaration of Helsinki, and met legal requirements (including ethical and radiation protection regulations). According to Swedish law (SFS 2003:460), a study regarded as quality work does not need formal approval from local research ethics committee.

### Myocardial perfusion scintigraphy

The MPS tests were performed per clinical routine in the departments, using a 2-day gated stress/gated rest protocol in Malmö and Odense while a 2-day non-gated stress/gated rest protocol was used in Gothenburg. The protocols used at the different sites are described in Table 1.

**Table 1:** Imaging protocols used at the different sites

	**Gothenburg**	**Malmö**	**Odense**
Radiopharmaceutical	Sestamibi	Tetrofosmin	Sestamibi/tetrofosmin
Administered activity (MBq)	600	600	800 to 1,000
Method of stress	Adenosine or maximal exercise	Adenosine or maximal exercise	Adenosine with light exercise
Time elapsed between tracer injection and measurement (min)	60	60	60
Camera system(s)	GE Infinia/Millenium	Siemens E.Cam	GE Ventri
Collimator	LEHR	LEHR	LEHR
*N* of measured projections	60 over 180° starting at 45° right anterior oblique	64 over 180° starting at 45° right anterior oblique	64 over 180° starting at 45° right anterior oblique
Acquisition time (second per projection)	40	20	20
Patient position	Supine	Supine	Supine
Pixel size after reconstruction (mm)	6.9	6.6	6.4
Reconstruction method	FBP	FBP	OSEM

LEHR, low energy high resolution; FBP, filtered back-projection; OSEM, ordered subset expectation maximization.

### Statistical methods

Descriptive statistics were performed according to data type; i.e., categorical variables were presented as frequencies and percentages, continuous variables were analyzed by mean, standard deviation, number or observations, minimum, median, maximum. Inter-observer agreement was investigated by means of Fleiss’ kappa [6,7] and respective 95% confidence intervals (95% CI) which were assessed using bootstrapping techniques [8]. Changes from first to second acquisition were assessed by McNemar’s test. Interrelationships between the technologists’ quality assessment and the physicians’ confidence in rating myocardial ischemia and infarction (measured on a 5-point scale) were explored by generalized logit regression model fitting in which the dependent variable was binary (being definitely sure of either existent or nonexistent ischemia/infarction versus indifference or possible existent or nonexistent ischemia/infarction). Independent variables were the technologists’ quality assessment and the acquisition number (first or second). The clustered nature of the data on a patient level (i.e., repeated evaluation of the same images by several technologists) was accounted for in order to use appropriate standard errors for the calculation of 95% CI for point estimates. Analyses were done for each physician separately. Receiver operating characteristic (ROC) curve analysis was performed on the technologists’ quality assessment to estimate the data-driven optimal threshold for maximizing the physicians’ confidence in rating myocardial ischemia and infarction. The optimal cutoff value was calculated as the point closest to the (0,1) point in the upper left corner of the ROC graph [9]. For the association between technologists’ and physicians’ assessments, only studies which included all assessments were included (a total of 111 studies). A sub-analysis was performed for the interrelationships between the technologists’ quality assessment and the physicians’ confidence in rating myocardial ischemia and infarction, for the different reconstruction methods (FBP, Gothenburg and Malmö and OSEM, Odense).

All of the hypothesis testing was done for exploratory purposes only without adjustment for multiple testing. Significance level was 5% (two-sided). All analyses were performed using SAS 9.1.3 (SAS Institute Inc., Cary, NC, USA) or Stata/MP 12.1 (StataCorp LP, College Station, Texas, USA).

## Results and discussion

### Quality assessment made by nuclear medicine technologists

The classifications made by the nuclear medicine technologists for the first and repeat acquisition are shown in Table 2. There was a statistically significant increase in image quality between the first and the repeat acquisition (1,256 cases of increased quality at the repeated study (66%), 134 cases of decreased quality at the repeated study (7%), and 510 cases of unchanged quality (27%); *P* < 0.0001).

**Table 2:** Cross-tabulation of quality assessment made by nuclear medicine technologists (first versus second acquisition)

**First acquisition**	**Second acquisition**					
	**1**	**2**	**3**	**4**	**5**	**Total**
**1**	54	90	114	68	51	377
**2**	72	265	345	193	173	1,048
**3**	8	34	88	98	84	312
**4**	1	4	6	19	40	70
**5**	1	0	2	6	84	93
**Total**	136	393	555	384	432	1,900

Improvements in quality from first to second acquisition are to be found above the main diagonal (i.e., in the upper right corner of the table).

The kappa values for the quality assessments done by the ten technologists were 0.36 (95% CI 0.31 to 0.41) and 0.40 (0.36 to 0.44) in the first run and second run, respectively, reflecting a fair agreement. The agreement was moderate in Gothenburg and Odense in both situations and fair in Malmö (Gothenburg = first run kappa 0.48 (0.40 to 0.57), second run 0.49 (0.43 to 0.56), Malmö = first run kappa 0.30 (0.24 to 0.37), second run 0.39 (0.34 to 0.45), Odense = kappa first run 0.40 (0.31 to 0.49), second run 0.47 (0.40 to 0.54).

### Interpretations made by physicians

The number of equivocal studies decreased when evaluating the repeated studies compared to the first studies for all physicians, both for the interpretations of ischemia and for infarction. For the first physician, the number of equivocal studies decreased from 16 at the first acquisition to 8 at the reacquisition for ischemia (*P* = 0.10) and from 9 to 8 for infarction (*P* = 0.99). For the second physician, the number of equivocal studies decreased from 50 to 21 for ischemia (*P* < 0.0001) and from 24 to 11 for infarction (*P* = 0.007). For the third physician, the numbers decreased from 40 to 21 for ischemia (*P* = 0.002) and from 21 to 17 for infarction (*P* = 0.59). Cross-tabulations of quality assessment with respect to ischemia and infarction made by the physicians for the first and repeat acquisition are shown in Table 3 (ischemia) and Table 4 (infarction).

**Table 3:** Cross-tabulation of diagnosis with respect to ischemia made by physicians (first versus second acquisition)

**First acquisition**	**Second acquisition**					
	**1**	**2**	**3**	**4**	**5**	**Total**
**1**	56	15	4	2	0	77
**2**	36	45	7	17	7	112
**3**	10	45	31	11	9	106
**4**	8	17	6	9	10	50
**5**	4	4	2	10	25	45
**Total**	114	126	50	49	51	390

1 = no ischemia; 3 = equivocal; 5 = certain ischemia.

**Table 4:** Cross-tabulation of diagnosis with respect to infarction made by physicians (first versus second acquisition)

**First acquisition**	**Second acquisition**					
	**1**	**2**	**3**	**4**	**5**	**Total**
**1**	112	27	4	0	0	143
**2**	30	58	11	9	1	109
**3**	4	22	14	11	3	54
**4**	2	7	5	11	16	41
**5**	0	2	2	4	35	43
**Total**	148	116	36	35	55	390

1 = no infarction; 3 = equivocal; 5 = certain infarction.

### Interrelationships between the technologists’ quality assessment and the physicians’ confidence in rating myocardial ischemia and infarction

We modeled the influence of the technologists’ quality assessment on the physicians’ confidence in rating ischemia and infarction (very sure versus less sure or equivocal). With respect to ischemia, one unit increase in the technologists’ quality assessment led on average to a 1.3- to 1.9-fold statistically significant increased chance of the physician being very confident with his/her evaluation (either absence or presence of ischemia), whereas the acquisition number did not have a statistically significant influence on the physicians’ confidence (Table 5). Regarding infarction, only for one physician did one unit increase in the technologists’ quality assessment lead on average to a 1.4-fold statistically significant increased chance of the physician being very confident with his/her evaluation (95% CI, 1.1 to 1.8, *P* = 0.01). Again, the acquisition number did not have a statistically significant influence on the physicians’ confidence. Table 6 shows the results from the subgroup analysis on Odense patients (OSEM), and Gothenburg and Malmö patients (FBP). Table 7 shows the movements between the quality classes from the first to the repeat acquisition by reconstruction method. Movements are shown by incremental improvement and worsening from the first to the second acquisition (e.g. ‘improvement by one point’) and were comparable for FBP and OSEM.

**Table 5:** Results from generalized logit regression model fitting

**Endpoint**	**Physician**	**Technologists' quality assessment**				**First or second acquisition**			
		**Odds ratio**	**95% CI**		***P *** **value**	**Odds ratio**	**95% CI**		***P *** **value**
Ischemia	1	1.9	1.4	2.6	<0.0001	1.1	0.6	2.0	0.86
	2	1.8	1.4	2.3	<0.0001	1.5	0.8	2.8	0.23
	3	1.3	1.03	1.6	0.03	1.0	0.5	1.8	0.93
Infarction	1	1.1	0.8	1.5	0.61	0.9	0.5	1.8	0.76
	2	1.4	1.1	1.8	0.01	1.2	0.7	2.1	0.48
	3	1.1	0.8	1.4	0.58	1.0	0.6	1.9	0.94

**Table 6:** Results from generalized logit regression model fitting—subgroup analysis

**Subgroup**	**Endpoint**	**Physician**	**Technologists' quality assessment**				**First or second acquisition**			
			**Odds ratio**	**95% CI**		***P *** **value**	**Odds ratio**	**95% CI**		***P *** **value**
OSEM^a^	Ischemia	1	1.2	0.4	3.5	0.80	2.3	0.3	22.1	0.49
		2	2.0	0.7	5.6	0.21	0.4	0.1	2.7	0.38
		3	1.6	0.7	3.7	0.32	0.4	0.1	3.1	0.36
	Infarction	1	0.9	0.3	2.6	0.86	0.6	0.1	5.6	0.67
		2	1.5	0.5	5.1	0.51	0.7	0.1	3.6	0.67
		3	1.1	0.4	3.5	0.86	3.3	0.3	37.7	0.33
FBP^b^	Ischemia	1	1.9	1.4	2.6	<0.0001	0.9	0.5	1.8	0.83
		2	1.9	1.4	2.5	<0.0001	1.8	0.9	3.8	0.10
		3	1.3	1.02	1.7	0.03	1.1	0.6	2.2	0.80
	Infarction	1	1.0	0.7	1.5	0.86	0.9	0.5	1.9	0.79
		2	1.4	1.0	1.8	0.03	1.3	0.7	2.2	0.44
		3	1.1	0.8	1.4	0.60	0.9	0.5	1.7	0.77

^a^OSEM, Odense patients; ^b^FBP, Gothenburg and Malmö patients.

**Table 7:** Movements between the classes from the first to the second acquisition by reconstruction method

**Change from first to second acquisition**	**Reconstruction method**		**Total (%)**
	**FBP (%)**	**OSEM (%)**	
Improvement by 4 points	29 (2.5)	22 (3)	51 (2.7)
Improvement by 3 points	156 (13.4)	85 (11.6)	241 (12.7)
Improvement by 2 points	226 (19.4)	165 (22.5)	391 (20.6)
Improvement by 1 point	345 (29.6)	228 (31.1)	573 (30.2)
No change	340 (29.1)	170 (23.2)	510 (26.8)
Worsening by 1 point	62 (5.3)	56 (7.6)	118 (6.2)
Worsening by 2 points	8 (0.7)	6 (0.8)	14 (0.7)
Worsening by 3 points	-	1 (0.1)	1 (0.05)
Worsening by 4 points	-	1 (0.1)	1 (0.05)
Total	1,166 (100)	734 (100)	1,900 (100)

FBP, filtered back-projection; OSEM, ordered subset expectation maximization.

**Figure 3:**
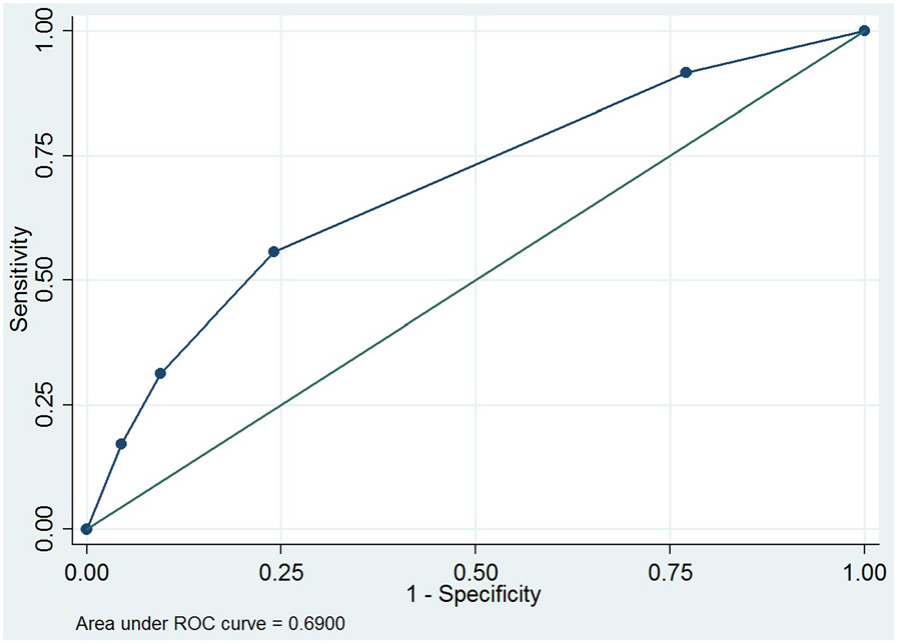
**ROC curve for physician 1 with respect to ischemia.** The dots, starting from the lower left corner, in the ROC curve, represent a cutoff of more than 5, between 4 and 5, between 3 and 4, between 2 and 3, between 1 and 2, and less than 1.

ROC analyses revealed that for both endpoints (ischemia, infarction) and all physicians, the optimal cutoff point for performing a repeat acquisition was a technologists’ quality assessment between categories 2 and 3. With respect to ischemia, the area under the ROC curve varied from 0.59 to 0.71 between the three physicians; regarding infarction, it varied from 0.53 to 0.62 (data not shown). Figure 3 shows the ROC curve for physician 1 with respect to ischemia. The optimal cutoff value, i.e., the point closest to the (0,1) point in the upper left corner of the ROC graph, relates to a cutoff between categories 2 and 3 in the technologists’ rating. This means that the technologists’ scores equal to or larger than 3 were the best predictor for strong confidence in the physicians’ rating; while technologists’ scores of 1 or 2 were the best predictor for limited confidence in the physicians’ rating, as compared to other possible cutoff points for the technologists’ scores.

## Discussion

In this study we wanted to determine rules of thumb for when reacquisition is useful due to high extra-cardiac uptake adjacent to the heart, i.e., when interpretation of the studies is affected by poor image quality. The European Council on Nuclear Cardiology (joint group of the European Association of Nuclear Medicine and of the European Association of Nuclear Medicine and of the European Society of Cardiology) wrote in their European procedural guidelines from 2005 that MPS images should be reviewed for the presence of extra-cardiac ‘hot spots’ so close to the left ventricle that they interfere with reconstruction and processing (e.g., lung, liver, gall bladder, and muscle) and to consider whether repeated acquisition is relevant after a time interval [10]. However, it is not clear from the guidelines when extra-cardiac activity interferes with reconstruction and processing, i.e., affects image quality, and should be repeated.

In the present study, we found significantly higher quality in the repeat acquisition than in the first. Since the technologists were not aware of the order of the acquisitions, we can conclude that it is useful to repeat the acquisition when high extra-cardiac activity is present. We also found that for both ischemia and infarction, the optimal cutoff point for performing a repeat acquisition was a technologists’ quality assessment between categories 2 and 3. Thus, based on this study, reacquisition is recommended when the intensity of the extra-cardiac uptake is equal to or higher than the cardiac uptake when there is no separation between the extra-cardiac uptake and the inferior cardiac wall, and when the intensity of the extra-cardiac uptake is higher than the cardiac uptake when there is a separation between the extra-cardiac uptake and the inferior wall of less than one cardiac wall. The relationship between the technologists’ quality assessment and the physicians’ confidence in assessing ischemia was stronger than the relationship between the technologists’ quality assessment and the physicians’ confidence in assessing infarction. The subgroup analyses for the interrelationships between the technologists’ quality assessment and the physicians’ confidence rating in myocardial ischemia and infarction revealed that the main results were driven by the subgroup of patients from Gothenburg and Malmö, as 95 out of 111 patients were from Gothenburg and Malmö. The subgroup results relating to Odense patients underlay larger variation due to the limited number of patients, leading to wider confidence intervals, but showed the same tendencies as the main result.

In a recent simulation study from our group, similar results were found [11]. In that study, MPS with inferior defects of different sizes as well as extra-cardiac uptake with different intensities and distances from the inferior cardiac wall were Monte Carlo simulated. The EXINI heart^TM^ software was used to evaluate the extent and severity of the defects. The conclusion from the study was that reacquisition should be considered when the extra-cardiac intensity is equal to the myocardium intensity at a distance of 0.5 cardiac wall thickness, or extra-cardiac intensity twice the myocardium intensity at a distance of 1 cardiac wall thickness. For extra-cardiac intensities that are half (or less than half) the intensity of the myocardium, no reacquisition is necessary. Previous studies have investigated the impact of high extra-cardiac uptake, especially high hepatic uptake, on the quantitative analysis of MPS images, using static phantoms [12-14]. Heller et al. [13] concluded that the addition of attenuation correction in the presence of extra-cardiac activity can have complex effects on maximum likelihood reconstruction with nonuniform attenuation correction, which depends on the amount of extra-cardiac activity and pattern of attenuation. It should be noted that we only used nonattenuation-corrected images in the present study, and that the results might be different when using attenuation-corrected images.

The strengths of the present study are the large number of patients included, that it was a multi-center study and that we have used several technologists and physicians for assessments of image quality and image interpretation. There were differences in the assessments made by both technologists and physicians, which is true for all diagnostic examinations. Thus, this study reflects a more real clinical setting compared to simulation studies or single-center studies, which commonly uses fewer physicians/technologists that are more likely to agree on assessments.

Several previous studies have investigated the number of repeated studies with regard to time from injection to image acquisition. Lyngholm et al. [15] studied the effect of time and food on upper abdominal activity in ^99m^Tc-tetrofosmin MPS. They graded each study on a 5-point semiquantitative scale. However, no real rule as when reacquisition was necessary was given in that study. In a study by Giorgetti et al. [16], the feasibility of early-image tetrofosmin protocol was investigated. Two observers visually analyzed the quality of the images using a 4-point scale, without further describing the criteria for the assessment.

In our study, the color scale used was the one used routinely in Gothenburg. The technologists from Gothenburg who evaluated the images were more familiar with the color scale used than the technologists from Malmö and Odense. Also, in Malmö, four technologists assessed image quality as compared to three from Gothenburg and Odense. This could partly explain the differences in kappa values for different sites with regard to quality assessment. There were 84 evaluations of 5, i.e., no extra-cardiac activity at all, in both first and second acquisition, which could be explained by reacquisition performed due to movement alone in at least six patients. One of the physicians assessed far fewer studies as ‘equivocal’ than the other two. This could partly be explained by that physician being more used to the EXINI heart™ software package which was used for image interpretation.

### Limitations of the study

The paper should be considered in the light of some limitations. We were not able to verify the diagnosis made by the physicians with an independent reference technique or follow-up of the patients. Also, different protocols and reconstruction parameters were used at the different study sites. Another limitation is that no attenuation-corrected images were available for analysis. No analysis of high extra-cardiac uptake resulting in decreased activity in the adjacent myocardium due to FBP was performed.

## Conclusion

Reacquisition is recommended when the intensity of the extra-cardiac uptake is equal to or higher than the cardiac uptake when there is no separation between the extra-cardiac uptake and the inferior cardiac wall and when the intensity of the extra-cardiac uptake is higher than the cardiac uptake when there is a separation between the extra-cardiac uptake and the inferior wall of less than one cardiac wall. We also found that the relationship between the technologists’ quality assessment and the physicians’ confidence in assessing ischemia was stronger than the relationship between the technologists’ quality assessment and the physicians’ confidence in assessing infarction.

## Competing interests

LE is a stockholder of EXINI Diagnostics AB, Lund, Sweden and KN is also employed by this company.

## Authors’ contributions

AJ, ML, and ET designed the study and interpreted all images. ET drafted the manuscript; AJ and ML revised the manuscript. OG helped with the design of the study, performed the statistical analysis, and contributed to the writing of the manuscript. LE and PW helped in the design of the study and revised the manuscript. LJ, GH, BJ, MSR, LLJ, and CO helped with the design of the study, assessed the image quality, and revised the manuscript. DM helped with the design of the study, provided insights into physics, and revised the manuscript. KN helped with the design of the study and organization/setup of all images. All authors read and approved the final version of the manuscript.

## Authors’ information

Allan Johansen and Milan Lomsky shared first authorship.
